# Malaria-Transmitting Vectors Microbiota: Overview and Interactions With *Anopheles* Mosquito Biology

**DOI:** 10.3389/fmicb.2022.891573

**Published:** 2022-05-20

**Authors:** Oswald Y. Djihinto, Adandé A. Medjigbodo, Albert R. A. Gangbadja, Helga M. Saizonou, Hamirath O. Lagnika, Dyane Nanmede, Laurette Djossou, Roméo Bohounton, Pierre Marie Sovegnon, Marie-Joel Fanou, Romuald Agonhossou, Romaric Akoton, Wassiyath Mousse, Luc S. Djogbénou

**Affiliations:** ^1^Tropical Infectious Diseases Research Centre (TIDRC), University of Abomey-Calavi, Cotonou, Benin; ^2^Regional Institute of Public Health, University of Abomey-Calavi, Ouidah, Benin

**Keywords:** *Anopheles*, mosquito, microbiota, malaria, insecticide resistance

## Abstract

Malaria remains a vector-borne infectious disease that is still a major public health concern worldwide, especially in tropical regions. Malaria is caused by a protozoan parasite of the genus *Plasmodium* and transmitted through the bite of infected female *Anopheles* mosquitoes. The control interventions targeting mosquito vectors have achieved significant success during the last two decades and rely mainly on the use of chemical insecticides through the insecticide-treated nets (ITNs) and indoor residual spraying (IRS). Unfortunately, resistance to conventional insecticides currently being used in public health is spreading in the natural mosquito populations, hampering the long-term success of the current vector control strategies. Thus, to achieve the goal of malaria elimination, it appears necessary to improve vector control approaches through the development of novel environment-friendly tools. Mosquito microbiota has by now given rise to the expansion of innovative control tools, such as the use of endosymbionts to target insect vectors, known as “symbiotic control.” In this review, we will present the viral, fungal and bacterial diversity of *Anopheles* mosquitoes, including the bacteriophages. This review discusses the likely interactions between the vector microbiota and its fitness and resistance to insecticides.

## Introduction

Malaria remains the most challenging tropical disease caused by parasites of the genus *Plasmodium*, transmitted through the bite of infected female *Anopheles* mosquitoes [Müller, 2011; World Health Organization [WHO], 2020]. This disease prompts an intense perilous illness and represents a prominent well-being danger in the most affected regions. In sub-Saharan Africa, where the highest number of malaria cases and mortality rates are recorded, this illness leads to a life-threatening condition, especially among children under 5 years old and pregnant women [World Health Organization [WHO], 2020].

Four components are involved in the human malaria transmission system: (i) the protozoan parasite *Plasmodium*, (ii) the human host, (iii) the mosquito vector and (iv) a given environment (Castro, 2017). Among the strategies developed to control this disease, targeting the vector has significantly reduced malaria incidence across Africa [Bhatt et al., 2015; World Health Organization [WHO], 2020]. The current vector control programs rely mainly on the use of chemical insecticides through the insecticide-treated nets (ITNs) with pyrethroids and the indoor residual spraying (IRS) with organophosphates and carbamates. The purpose of these conventional tools is to reduce vector density below the threshold required for transmission or to prevent human-vector contact (Karunamoorthi, 2011). Mosquitoes belonging to the genus *Anopheles* are among the most important malaria vectors in endemic regions (Hay et al., 2010). In African countries, the dominant *Anopheles* vectors of human malaria include *Anopheles gambiae*, *Anopheles arabiensis*, *Anopheles coluzzii* and *Anopheles funestus* (Battle et al., 2012; Sinka et al., 2012; Coetzee et al., 2013) as well as the recently confirmed urban environment species *Anopheles stephensi* (Sinka et al., 2020). Unfortunately, the resistance against conventional insecticides currently used in public health is spreading in the natural mosquito populations. Indeed, there is an increasing number of evidence of insecticide resistance in malaria-transmitting vectors, and this phenomenon is threatening the success of malaria vector control programs (Mekuriaw et al., 2019).

Furthermore, African countries are taking a heavy toll on the Covid-19 pandemic. Indeed, this pandemic emerging from China by the end of December 2019 accounts for 6.3 million cases and 152,927 deaths in the entire African continent by August 12, 2021 [Salyer et al., 2021; World Health Organization [WHO], 2021]. Malaria and Covid-19 can have common clinical manifestations, including fever, tiredness and acute onset headache, leading to a misdiagnosis of malaria for Covid-19 and vice versa (Hussein et al., 2020). In addition, the Covid-19 is hampering the mass distribution of ITNs (Hussein et al., 2020). These situations might hinder the long-term global technical strategy of the World Health Organization (WHO) to reduce malaria case incidence from 222 (in 2021) to 23 per 1,000 populations by 2030, toward malaria elimination [World Health Organization [WHO], 2020]. To reach malaria elimination, it appears necessary to design and implement innovative and environment-friendly approaches to control malaria vectors (Gabrieli et al., 2021).

In such a situation, the mosquito microbiota has by now given rise to the expansion of innovative control tools, such as the use of endosymbionts known as “symbiotic control” (Ricci et al., 2012; Gabrieli et al., 2021). However, before implementing this control measure in natural *Anopheles* mosquito populations, it will be helpful to understand the bacterial diversity in these vectors and their interactions with their host. The present review is designed to provide an overview of the *Anopheles* microbiota and discuss the potential implications for the vector fitness, immune response and resistance to insecticides.

## Viruses of *Anopheles* Mosquitoes Microbiota

Mosquitoes often harbor a diverse and dynamic viral composition. Since mosquito-pathogenic microbes can be used for mosquito control (Huang et al., 2020), a better understanding of the natural and acquired viral communities infecting directly *Anopheles* mosquitoes cells, is expected to provide a background for developing novel biological tools for malaria control in endemic areas.

### The Virome Diversity

Studies have analyzed the virome of different *Anopheles* mosquitoes and have shown variations in viruses diversity and abundance between the mosquito species (Nanfack Minkeu and Vernick, 2018). Previous studies reported that *Anopheles gambiae* and *Anopheles funestus* mosquitoes ensure the biological transmission of the o’nyong-nyong arbovirus (ONNV) (Fauver et al., 2016; Nanfack Minkeu and Vernick, 2018; Belda et al., 2019). ONNV is the only known human pathogenic alphavirus with *Anopheles* vectors and is responsible for an epidemic febrile polyarthralgia. The symptoms are headaches, pruritic rash, lymphadenopathy, and conjunctivitis (Barrett and Weaver, 2012). Another virus, the densonucleosis virus (AgDNV) belonging to the Parvoviridae family (subfamily Densovirinae) was found to infect and disseminate in *An. gambiae* mosquitoes (Ren et al., 2008). The newly characterized AgDNV was demonstrated to be favorably transmissible to *An. gambiae* larvae; to spread to adult tissues and vertically transmitted to the offspring (Ren et al., 2008). Therefore, AgDNV represents a valuable tool for viral paratransgenesis (the genetic manipulation of mosquito symbiotic microorganisms) for malaria vector control. However, within *Anopheles* hosts, virus infection and replication could have significant side effects on several physiological traits of their bearers.

Overall, more than fifty different virus species belonging to at least thirteen main genera (*Almendravirus*, *Alphavirus*, *Cripavirus*, *Cypovirus*, *Densovirus*, *Flavivirus*, *Iridovirus*, *Mononegavirus*, *Orbivirus*, *Orthobunyavirus*, *Phlebovirus*, *Poxvirus*, and *Totivirus*) were found infecting diverse *Anopheles* mosquito species in the tropical countries of the world (virus species and *Anopheles* species infected were reviewed in Nanfack Minkeu and Vernick, 2018). Different other viruses are capable of infection, dissemination and transmission in *Anopheles* mosquitoes (see details in Table 1). Recently two novel viruses corresponding to emergent clades of insect-specific negative-strand single RNA viruses, *Orthophasmavirus* (Bunyavirales) and *Anphevirus* (Mononegavirales) were found in *Anopheles* mosquitoes. *Orthophasmavirus* was discovered in *An. triannulatus* (Scarpassa et al., 2019) and in *Anopheles lutzi* (da Silva Neves et al., 2021), while *Anphevirus* was found in *An. marajoara*, and *An. darlingi* (Scarpassa et al., 2019). In addition, another metagenomic sequencing work revealed ten other viruses infecting *An. sinensis* namely *Culex Bunyavirus* 1 (Phenuiviridae), *Wutai mosquito phasivirus* (Phenuiviridae), *Wuhan Mosquito Virus 6* (Orthomyxoviridae), *Hubei virga-like virus 1* (Tymoviridae), *Yongsan picorna-like virus 4* (Iflaviridae), *Wuhan Mosquito Virus 9* (Rhabdoviridae), *Culex Y virus* (Birnaviridae), *Hubei virga-like virus 21* (Tymoviridae) *Hubei picorna-like virus 58* (Tymoviridae), and *Culex mononega-like virus 1* (Virgaviridae) (He et al., 2021). Furthermore, the porcine parvovirus (PPV) belonging to the genus *Parvovirus* (sub-family Parvovirinae, family Parvoviridae), was also identified in *Anopheles* mosquitoes including *An. sinensis* (Shi et al., 2015; Xia et al., 2018; Hameed et al., 2020).

**TABLE 1:** Viruses capable of infection, dissemination and transmission in *Anopheles* mosquitoes.

Species name	Genus	Subfamily	Family	Major host	*Anopheles* carrier	Mosquito infectivity	References
*O’nyong nyong virus*	*Alphavirus*	Togavirinae	Togaviridae	Insect	*Anopheles rufipes, Anopheles funestus, Anopheles gambiae* and *Anopheles coustani*	Infection, dissimilation and transmission to subsequent generations	Belda et al., 2019
*Densonucleosis virus (AgDNV)*	*Brevidensovirus*	Densovirinae	Parvoviridae	Insect	*Anopheles gambiae*	Infection, dissimilation and transmission to subsequent generations	Ren et al., 2008
*Culex theileri flavivirus* (CTFV)	*Flavivirus*	Flavivirinae	Flaviviridae	Insect	*Anopheles vagus*	Infection and dissimilation	Sadeghi et al., 2017
*Torque tenosus virus1* (TTSuV1)	*Iotatorquevirus*	Anellovirinae	Anelloviridae	Vertebrate	*Anopheles sinensis*	Infection and dissimilation	Shi et al., 2015
*Yunnan orbivirus* (YOUV)	*Orbivirus*	Sedoreovirinae	Reoviridae	Vertebrate	*Anopheles vagus*	Infection and dissimilation	Sadeghi et al., 2017
*Batai virus* (BATV)	*Orthobunyavirus*	-	Bunyaviridae	Vertebrate	*Anopheles maculipennis*	Infection and dissimilation	Jöst et al., 2011; Huhtamo et al., 2013

These data show a high diversity of viral communities carried by *Anopheles* mosquitoes, which may have the potential to infect a wide range of hosts. Further studies are required to characterize these viruses species in terms of prevalence, pathogenicity, and transmission to the vertebrate host during blood-feeding.

### Functions of the Virome

To date, to our knowledge, no study has provided the specific function of viruses in *Anopheles* vectors. Since *Anopheles* mosquitoes are the primary vectors of malaria parasites in endemic countries, coinfection of viruses and *Plasmodium* could occur in the same mosquito vector. It is then possible that a virus infection could suppress other viruses’ replication or block the *Plasmodium* transmission. Thus, a better understanding of the virus-mosquito interactions requires detailed and specific investigations.

## Fungi of *Anopheles* Mosquitoes Microbiota

Among the wide range of pathogens capable of infecting *Anopheles* mosquitoes, there are poor reports on fungi species in the natural core microbiota of mosquito populations. Unlike bacteria, viruses, and *Plasmodium* parasites that need to be ingested by the mosquitoes before being transmitted to the vertebrate hosts, fungi infect mosquito hosts through the cuticle and afterward proliferate in the hemolymph (Mannino et al., 2019).

### Diversity of the Fungal Community

The interactions between entomopathogenic or non-entomopathogenic fungi and mosquitoes remain not well understood. Previous studies have found an *Ascomycete* fungus, *Penicillium chrysogenum*, a non-entomopathogenic species in the midgut of field-caught *An. gambiae* mosquitoes (Angleró-Rodríguez et al., 2016). In *An. gambiae* maintained in semi-field conditions, several other taxa were found at different developmental stages (larvae, pupae and adult) and in different tissues (ovary, midgut, body carcass). These fungi isolates included *Hyphopichia burtonii*, *Hyphopichia sp*., *Penicillium georgiense, Periconia* sp. *Leptosphaerulina chartarum, Cladosporium cladosporioid, Hasegawazyma lactos, Epicoccum* sp., *Alternaria alternate*, and *Lichtheimia hyalospora* (Nattoh et al., 2021). Some yeast isolates, including *Meyerozyma guilliermondii*, *Rhodotorula glutinis*, were also identified in the guts of laboratory strains of *An. gambiae* with *M. guilliermondii* also found in *An. stephensi* gut (Bozic et al., 2017). Most importantly, the opportunistic pathogen *Candida parapsilosis*, was also found in the gut of both *An. gambiae* and *An. stephensi* strains. In addition, *C. parapsilosis* was found at all developmental stages and in adult male and female guts and reproductive tissues (Bozic et al., 2017). Another yeast species *Wickerhamomyces anomalus*, was found to colonize immature stages (larvae and pupae) and adults of *An. stephensi* at different ages (Ricci et al., 2011). Like the yeasts reported by Bozic et al. (2017), *W. anomalus* was also shown to be localized in the midgut of both male and female *An. stephensi* reproductive systems (Ricci et al., 2011). However, fungi of the genus *Aspergillus* have been observed in the midgut of field-collected *An. stephensi* larvae but not in the adult mosquitoes (Tajedin et al., 2009), suggesting that *Aspergillus* might play a specific role at the larval stage in this mosquito species.

Some fungi species infecting mosquito vectors and their transmission to the subsequent developmental stages suggest a transstadial transmission, which could shape adult mosquito’s physiology and likely female insects’ susceptibility to the malaria parasite infection. Therefore, further works are needed to thoroughly understand the mosquito-mycobiota interactions.

### Functions of the Fungal Community

*Anopheles* mosquitoes are colonized by a huge range of fungal microorganisms that may affect mosquito biology and vectorial capacity. According to the mosquito-mycobiota relationships, most fungi species are considered non-pathogenic (in commensal or symbiotic relationships) (Steyn et al., 2016) while some are pathogenic (developing parasitic interaction with mosquitoes) (Tawidian et al., 2019). Broad knowledge of the functions of fungi with the fitness of their mosquito host, as well as interactions with transmitted pathogens (especially *Plasmodium* parasites), is henceforth an important factor in the development and implementation of novel malaria vector control tools.

Although there are fewer reports on fungal species isolated from the natural microbiome community of mosquito populations, entomopathogenic fungi capable of experimentally infecting *Anopheles* vectors have been extensively studied (Bukhari et al., 2010; Howard et al., 2011; Valero-Jiménez et al., 2014). Indeed, it has been demonstrated that after topical infection, the entomopathogenic fungus *Beauveria bassiana* can interact with the gut bacteria in *An. stephensi* to accelerate the death of the mosquitoes (Wei et al., 2017). Both laboratory and wild-caught *An. arabiensis* were found susceptible to *Beauveria bassiana* infection regardless of their insecticide susceptibility (Kikankie et al., 2010). Using fungal suspensors, the authors found that exposure to *B. bassiana* spores reduced significantly the longevity of all mosquito colonies (Kikankie et al., 2010). Wild *An. arabiensis*, *An. gambiae* and *An. funestus* treated with mineral oil formulations of *B. bassiana* also resulted in a reduced lifespan of mosquitoes (Mnyone et al., 2012). Significant reduction in *An. gambiae* survival was also observed when mosquitoes were exposed to co-formulations of both *M. anisopliae* and *B. bassiana* fungus (Mnyone et al., 2009). The same trend was reported in *An. gambiae* exposed to dried conidia of *M. anisopliae* (Scholte et al., 2003) and in both *An. gambiae* and *An. funestus* exposed to low doses of conidia by direct contact (Farenhorst et al., 2008; Rhodes et al., 2018). *M. anisopliae* were able to infect wild *An. gambiae s.l.* and reduce mosquito longevity upon contact on *M. anisopliae-*impregnated black cotton sheets (Scholte et al., 2005). In laboratory conditions, it was shown that following mosquitoes infection, these fungi species can autodisseminate between the vectors through mating (Scholte et al., 2004). In addition, it was shown that age and blood-feeding status did not affect mosquito susceptibility to fungal infection (Mnyone et al., 2011). These findings are interesting as they could enhance the propagation of the fungal infection in mosquito populations when the biological malaria vector control based on these pathogens is implemented. However, further investigations are required to evaluate the autodissemination potential of the fungal community in the field populations of *Anopheles* vectors. The presence and the dissemination of some fungi into the reproductive organs of both male and female mosquitoes may suggest their potential involvement in mosquito reproduction and their likely vertical transmission (Ricci et al., 2011). Other mosquito life-history traits such as blood-feeding, flight ability, fecundity (Scholte et al., 2006; Blanford et al., 2011; Ondiaka et al., 2015); and host-seeking behavior (George et al., 2011) were also reported to be negatively affected following *B. bassiana* and *M. anisopliae* exposure. All these findings highlight the potential of fungi as biological control agents for malaria vector control.

Other fungi have been shown to contribute to the pathogen’s development in mosquitoes. However, it was reported that the presence of *Pe. chrysogenum* in the *An. gambiae* midgut does not affect mosquito survival, and it increases the mosquito susceptibility to *Plasmodium* infection by suppressing the host immune system (Angleró-Rodríguez et al., 2016). Conversely, *Leptosphaerulina* sp. was shown to activate the host immune system to induce melanization (production of melanin deposits) on the fat body in *An. gambiae* (Nattoh et al., 2021). Further knowledge regarding the interactions between host and pathogens is required to better understand how these fungi affect longevity and other mosquito life-history traits.

## The Bacterial Community and Its Associated Bacteriophages in *Anopheles* Mosquitoes

During the aquatic developmental period, bacteria constitute one of the major sources of nutrition for mosquito larvae (Walker et al., 1988). Numerous studies have demonstrated that mosquitoes host huge bacterial communities that vary according to the mosquito sex, developmental stage, and living environment (Minard et al., 2013). Endosymbionts have been proposed as a promising candidate to develop paratransgenesis approaches (Coutinho-Abreu et al., 2010). This prompts the urgent need to deeply understand the bacterial spectrum in malaria vectors.

### Diversity of the Bacterial Community

As *Anopheles* mosquitoes are the definitive hosts responsible for malaria parasite transmission, the composition and diversity of the bacterial communities they host apart from *Plasmodium* species need to be taken into account. Several studies revealed the hugeness of bacterial diversity in *Anopheles* vectors (Table 2). It was reported the large presence of the uncultured *Paenibacillaceae* in male *An. stephensis* and *Serratia marcescens* in females and larvae individuals. The authors also reported that, unlike field-collected mosquitoes, *Serratia marcescens* and *Cryseobacterium meninqosepticum* bacteria were abundant in the laboratory-reared *An. stephensis* strain (Rani et al., 2009).

**TABLE 2:** Common bacteria microbiota infecting *Anopheles* species mosquitoes.

Bacterial family	Bacterial species	Mosquito species	References
-	*Bacteroidetes*	*An. gambiae*	Wang et al., 2011
*-*	*Proteobacteria*	*An. gambiae;* An. funestus	Wang et al., 2011; E Silva et al., 2021
Acetobacteraceae	*Asaia* sp.; *Asaia bogorensis*	*An. stephensi*; *An. gambiae*	Favia et al., 2007; Damiani et al., 2010; Wang et al., 2021
Aeromonadaceae	Aeromonas hydrophila	An. arabiensis	E Silva et al., 2021
Bacillaceae	*Bacillus* sp.	*An. darlingi*	Rocha et al., 2021
Bifidobacteriaceae	*Bifidobacterium*	*An. lesteri*	Feng et al., 2021
Brevibacteriaceae	*Brevibacterium* sp.	*An. darlingi*	Rocha et al., 2021
Burkholderiaceae	*Burkholderia*	*An. darlingi*	Arruda et al., 2021
Comamonadaceae	*Comamonas* sp.	*An. arabiensis*	Cirimotich et al., 2011; Bahia et al., 2014
*Cyanophyceae*	*Cyanobacteria*	*An. gambiae*	Wang et al., 2011
Enterobacteriaceae	*Cedecea* sp.	*An. darlingi*	Arruda et al., 2021
	*Enterobacter* sp.; Enterobacter cloacae	*An. darling*; *An. gambiae*; *An. funestus*; *An. arabiensis*	Arruda et al., 2021; Rocha et al., 2021; Ezemuoka et al., 2020; E Silva et al., 2021
	*Escherichia coli*	*An. lesteri*	Feng et al., 2021
	*Klebsiella* sp.	*An. darlingi*	Arruda et al., 2021; Rocha et al., 2021
	*Pantoea* sp.	*An. darlingi*; *An. arabiensis*	Arruda et al., 2021; Rocha et al., 2021; Cirimotich et al., 2011; Bahia et al., 2014
	*Serratia* sp.; *Serratia liquefaciens; Serratia* oryzae	*An. darling*; An. arabiensis; *An. stephensis; An. gambiae*; *An. arabiensis*	Arruda et al., 2021; E Silva et al., 2021; Rocha et al., 2021; Rani et al., 2009; Ezemuoka et al., 2020; Cirimotich et al., 2011; Bahia et al., 2014
	*Shigella* sp.	*An. lesteri*	Feng et al., 2021
Flavobacteriaceae	*-*	*An. gambiae*	Wang et al., 2011
Lachnospiraceae	*Blautia* sp.	*An. lesteri*	Feng et al., 2021
Microbacteriaceae	*Leucobacter* sp.	*An. darlingi*	Rocha et al., 2021
	*Microbacterium* sp.	*An. darlingi*	Rocha et al., 2021
Micrococcaceae	*Arthrobacter* sp.	*An. darlingi*	Rocha et al., 2021
Moraxellaceae	*Acinetobacter* sp.	*An. darling*; *An. arabiensis*	Arruda et al., 2021; Rocha et al., 2021; Cirimotich et al., 2011; Bahia et al., 2014
*Paenibacillaceae*	*Paenibacillus*	*An. stephensis*	Rani et al., 2009
Pseudomonadaceae	*Pseudomonas putida; Pseudomonas rhodesiae*	*An. dirus*; *An. arabiensis*	Feng et al., 2021; Cirimotich et al., 2011; Bahia et al., 2014
Rickettsiaceae	*Wolbachia* sp.	*An. maculatus*; *An. sinensis*; *An. funestus*	Wong et al., 2020; Baldini et al., 2014; Gomes et al., 2017; Ayala et al., 2019; Niang et al., 2018
Ruminococcaceae	*Faecalibacterium* sp.	*An. lesteri*; *An. dirus*	Feng et al., 2021
Staphylococcaceae	*Staphylococcus* sp.; Staphylococcus epidermidis; Staphylococcus hominis; *Staphylococcus ureilytica*	*An. darling*; *An. arabiensis*; *An. funestus*; *An. sinensis*	Arruda et al., 2021; E Silva et al., 2021; Gao et al., 2021
*Weeksellaceae*	*Cryseobacterium meninqosepticum*	*An. stephensis*	Rani et al., 2009
	*Elizabethkingia anophelis*	*An. arabiensis*; *An. darlingi*	Cirimotich et al., 2011; Bahia et al., 2014; E Silva et al., 2021; Rocha et al., 2021
Xanthomonadaceae	*Stenotrophomonas* sp.	*An. darlingi*	Rocha et al., 2021

Studies have characterized the bacterial communities in larvae, pupae and adults of *An. gambiae* reared in semi-natural habitats. It was shown that photosynthetic *Cyanobacteria* were predominant in the larval and pupal guts while *Proteobacteria* and *Bacteroidetes* were abundant in adults guts with core taxa of *Enterobacteriaceae* and *Flavobacteriaceae* (Wang et al., 2011). *Enterobacter cloacae* and *Serratia marcescens* were two predominant species among the bacteria isolated from *An. gambiae s.l.* (Ezemuoka et al., 2020). Fourteen bacteria species from eight different genera (*Staphylococcus, Burkholderia, Cedecea*, *Enterobacter, Klebsiella, Pantoea, Serratia*, and *Acinetobacter*) were identified in the feces of wild *An. darlingi* (Arruda et al., 2021). The most frequent species were members of the *Serratia* genus with *Serratia liquefaciens* and *Serratia marcescens* the major representative bacterial species (Arruda et al., 2021). It was reported in the malaria vectors *An. funestus* and *An. arabiensis*, the presence of a number of bacteria even weeks after mosquitoes’ preservation on silica or in RNAlater^®^ solution (E Silva et al., 2021). The authors found that the midgut of *An. arabiensis* was mainly colonized by *Bacteroidetes* and *Proteobacteria*, with the latter being the predominant one colonizing *An. funestus* midgut (E Silva et al., 2021). *Elizabethkingia* and *Serratia* were found in both *An. arabiensis* males and females, while *Serratia*, *Elizabethkingia* and *Aeromonas* were the dominant genera in males *An. arabiensis* (E Silva et al., 2021). The same authors discovered that at the species level, *Elizabethkingia anophelis*, *Serratia oryzae* and *Aeromonas hydrophila* were common bacteria between female and male *An. arabiensis*. In the preserved field-collected *An. arabiensis, Staphylococcus* was the dominant genus, with *Staphylococcus epidermidis* and *Staphylococcus hominis* present after 8 and 12 weeks of preservation (E Silva et al., 2021). In both *An. funestus* and *An. arabiensis, Enterobacter cloacae* was specific to females and *S. epidermidis* was specific to male mosquitoes (E Silva et al., 2021). It appears that the bacterial communities can be well preserved in *Anopheles* mosquitoes. However, it was reported that the bacterial composition and diversity in *An. gambiae s.l.* rely upon several factors such as season, geography and environmental variations (Akorli et al., 2016; Krajacich et al., 2018). In field-caught *An. arabiensis* from Zambia, seven bacterial species including *Comamonas sp*., *Acinetobacter sp., Pseudomonas putida, Pantoea sp., Pseudomonas rhodesiae, Serratia marcescens* and *Elizabethkingia anophelis* were isolated from their midgut (Cirimotich et al., 2011; Bahia et al., 2014).

In *Anopheles* mosquitoes from china, *Bifidobacterium*, *Faecalibacterium*, *Escherichia-Shigella*, and *Blautia* were significantly enriched in *An. lesteri*, wheras *Pseudomonas* and *Ruminococcaceae* were predominant in *An. dirus* and *Asaia* in *An. sinensis* (Feng et al., 2021). The genus *Asaia*, previously reported as the dominant bacterium in *An. stephensi* microbiota (Favia et al., 2007), was also found in all aquatic stages (eggs, L1–L4 larvae, pupae) as well as in the midgut, salivary glands and reproductive tissues of both laboratory-reared and filed-collected adult *An. gambiae* mosquitoes (Damiani et al., 2010).

Actinobacteria including *Arthrobacter sp.*, *Brevibacterium sp*., *Leucobacter sp*., and *Microbacterium sp*., were found in adults, larvae, pupae and eggs of *An. darlingi* (Rocha et al., 2021). The common genera including *Acinetobacter*, *Enterobacter*, *Klebsiella*, *Serratia*, *Bacillus*, *Elizabethkingia*, *Stenotrophomonas* and *Pantoea* were predominantly identified in this mosquito species (Rocha et al., 2021). The natural endosymbiont bacterium *Wolbachia* was found in diverse field-caught *Anopheles* species including *An. maculatus, An. sinensis* and other *Anopheles* species collected in Malaysia (Wong et al., 2020). The presence of *Wolbachia* in field populations of *An. gambiae* has also been reported in Burkina-Faso (Baldini et al., 2014); in Mali (Gomes et al., 2017); in a large number of *Anopheles* species from Gabon (Ayala et al., 2019); and in *An. funestus* populations in Senegal (Niang et al., 2018).

*Anopheles* species harbor huge microbial communities. However, the existence of a main bacterial community is still not clear (Romoli and Gendrin, 2018). The Gram-negative aerobic or facultative aerobic bacteria, mostly belonging to the families *Enterobacteriaceae* (*Serratia*, *Ewingella*, *Enterobacter* and *Klebsiella*), *Acetobacteraceae* (*Acetobacter* and *Asaia*) and *Flavobacteriaceae* (*Elizabethkingia* and *Chryseobacterium*) represent the bacterial genera commonly found in *Anopheles* mosquitoes (Gendrin and Christophides, 2013). An individual mosquito host variable and dynamic microbiota (Table 2). Such diversity in the bacterial composition could depend on environmental factors and individual history. Indeed, the implication of the environment in shaping mosquitoes was reported in previous studies (Boissière et al., 2012; Osei-Poku et al., 2012). Furthermore, it was shown that larval breeding water and adult mosquito sugar food contribute to midgut microbiota composition in mosquitoes (Saab et al., 2020). However, some authors suggest that genetic factors might be more important than environmental factors in influencing the divergence of mosquito microbiota across the different species (Feng et al., 2021). Some bacteria genera were suggested to be transmitted from females to offspring by likely vertical symbiont transmission mechanism *via* eggs (Damiani et al., 2010). Indeed, effective horizontal and vertical transmission routes of bacteria were already described in *An. stephensi* (Favia et al., 2007; Damiani et al., 2008).

### Functions of the Bacteria in *Anopheles* Mosquitoes

Although further studies are needed to investigate the role that bacteria play in *Anopheles* mosquitoes, previous works have attempted to provide more insights. Bacterial communities have been shown to impede *Plasmodium* development in *An. gambiae* (Beier et al., 1994). An association of distinct bacteria with the pyrethroid resistance in *An. gambiae* has been reported (Omoke et al., 2021). Bacteria of *Anopheles* vectors interfere with both the physiology and vector competence of their bearers. Several studies have revealed an overall inhibitory effect of the bacterial communities on *Plasmodium* parasites in *Anopheles* species. It was found that the intestinal bacterial communities can regulate the expression of the thioester-containing protein (TEP1) *via* an RNA interference (RNAi) mechanism to inhibit *P. yoelii* development in *An. dirus* (Wang et al., 2013). Other bacterial species, including *Escherichia coli* (strains H243, HS5); *Pseudomonas aeruginosa*; *Pseudomonas sutzeri*a; *Ewingella americana, Serratia marcescens*; *Xanthomonas malthophila*; *Cedecea lapagei*a; *Enterobacter cloacae*; *Enterobacter amnigenus, S. aureus*; *Comamonas* spp.; *Bacillus pumilus; Chromobacterium sp. Csp_P*, and *Methylobacterium* were reported to affect the development of *P. falciparum*, *P. vivax*, and *P. berghei* in *An. gambiae* (Dong et al., 2009; Meister et al., 2009; Cirimotich et al., 2011; Bahia et al., 2014; Ramirez et al., 2014; Tchioffo et al., 2016), *An. stephensi* (Pumpuni et al., 1993, 1996; Cirimotich et al., 2011; Bando et al., 2013)*, An. albimanus* (Gonzalez-Ceron et al., 2003) and *An. coluzzii* (Meister et al., 2009; Tchioffo et al., 2013). Another bacterial species of the genus *Serratia, S. ureilytica*, isolated from the midguts of wild *An. sinensis* mosquitoes have been shown to inhibit the development of *P. falciparum* or the rodent parasite *P*. *berghei* by producing a lipase that is lethal to the parasites at different developmental stages (Gao et al., 2021). Contrary, in *An. stephensi*, the bacterium *Asaia bogorensis*, was demonstrated to increase the midgut pH and subsequently promote the *Plasmodium berghei* gametogenesis by alkalizing the mosquito midgut (Wang et al., 2021). The genome of the *Asaia* strain isolated from *An. stephensi* was analyzed and it was found that this bacterial strain had the most predicted regulatory proteins, suggesting its ability to adapt to frequent environmental changes in the mosquito gut (Chen et al., 2021).

Furthermore, *Wolbachia* infection was shown to significantly reduce the prevalence and intensity of sporozoite infection in *An. gambiae s.l*. (Gomes et al., 2017). Besides the negative correlation with *Plasmodium* development, *Wolbachia* infections in natural *Anopheles* populations also affect egg-laying (Shaw et al., 2016). Reintroduction of *Pseudomonas putida*, *Pantoea sp.*, and *Serratia marcescens* into *An. arabiensis* through sugar feeding, resulted also in significant inhibition of *P. falciparum* infection, with *S. marcescens* and *P. putida* exhibiting the strongest parasite-blocking activity (Bahia et al., 2014). Experimental infection of *Chromobacterium violaceum* in insecticide-resistant *An. coluzzii* females was shown to significantly reduce mosquito survival, blood-feeding and to affect fecundity and hatching rate (Gnambani et al., 2020).

The biology of bacterial communities is an essential determinant for *Anopheles* mosquitoes’ fitness and resistance to insecticide molecules. During mosquito development, *Anopheles* mosquito larvae feed on microorganisms, organic matter and biofilm in aquatic habitats. Biofilm on the breeding site water surface is enriched with bacteria and provides nutrients to the larvae (Cansado-Utrilla et al., 2021). Some ingested bacteria in aquatic habitats colonize the mosquito’s gut and are transmitted to the adult stage (Wotton et al., 1997). The colonization of the host organism by the bacteria is mutually beneficial to both. Indeed, the host provides a stable, nutrient-rich environment, and the bacteria assist in digestion, protection from opportunistic pathogens, and immune system maturation (Kamada et al., 2013).

Modulating mosquito’s bacterial composition could shape the vector lifespan or the susceptibility to the existing classes of insecticides. Indeed, it was reported that bacteria depletion by using bactericidal antibiotics increased the longevity of susceptible *An. arabiensis* while bacterial supplementation increased insecticide tolerance in the resistant individuals (Barnard et al., 2019). On the contrary, other authors have shown that antibiotic mediated disorder of gut homeostasis leads to a decreased longevity in *An. arabiensis* (Debalke et al., 2019). Furthermore, dysbiosis in *An. gambiae s.l.* through antibiotics treatment resulted in reduced lifespan while the reintroduction of *Enterobacter cloacae* and *Serratia marcescens* in female mosquitoes did not affect the average fecundity but they positively affected hatching rates (Ezemuoka et al., 2020). However, microbiome dysbiosis using oxytetracycline has been shown to reduce the fecundity of *An. gambiae* bearing *kdr^R^* (L1014F) allele (Medjigbodo et al., 2021).

Overall, the molecular mechanisms involved in the interactions between *bacteria* and mosquito hosts, as well as between bacterial species of the mosquito microbiome remain not well understood. Further investigations are needed to investigate the mechanisms by which the vector longevity and fecundity are influenced by the gut bacterial community. Adult male mosquitoes obtain their nutrients from sugar, while both sugar and blood constitute the food sources for females. Consequently, the microbiome is involved in food digestion. Even though it was found in other mosquito species that *Enterobacteriaceae* is the main family of the gut microbiota at assimilating monosaccharides (Guégan et al., 2020), this is not yet characterized in *Anopheles* species. Therefore, the influence of the gut-associated microbiota on *Anopheles* vectors nutrition remains a field of interest not well explored and open for subsequent studies.

### Bacteriophages in Mosquito Bacterial Communities

Bacteriophages were discovered in 1915 and are viruses infecting bacteria and archaebacteria (Clokie et al., 2011). They are found in all biomes (Herridge et al., 2020), including mosquito vectors. Although some of these viruses are latent (the infection does not immediately result in cell death), there are virulent bacteriophages that only replicate through a lytic cycle to release new virions and kill their bacterial host (Chevallereau et al., 2022). There are also obligate lytic bacteriophages that are lethal to a specific bacteria genus of a particular species (de Jonge et al., 2019). Besides, lytic bacteriophages were already isolated from bacterial species, which can degrade insecticides. Indeed, three bacteriophages were isolated from bacteria belonging to the *Klebsiella* genus, bacteria associated with organophosphate insecticide resistance in *Anopheles albimanus* (Dada et al., 2018). These isolated bacteriophages belong to the Podoviridae family and have demonstrated strong lytic potential *in vitro* against *Klebsiella pneumoniae* strain ZS15 (Kupritz et al., 2021). Moreover, it was demonstrated that phage G, initially considered a bacteriophage of *Bacillus megaterium*, is a *Lysinibacillus* bacteriophage (González et al., 2020). *Lysinibacillus* is a pyrethroid-degrading taxon and bacteria genus associated with permethrin resistance in western Kenyan *An. gambiae* (Omoke et al., 2021).

The presence of bacteriophages in insecticide degrading bacteria of malaria vectors suggests that bacteriophages infection could be involved in mosquito bacterial composition regulation as they attack and kill bacteria. As a result, the isolation and the characterization of mosquito bacteriophages communities may lead to the identification of virulent bacteriophages that could be used to design biological alternative vector control strategies. However, investigations on the diversity and the composition of *Anopheles* mosquito’s bacteriophages communities are still lacking.

## The Interplay Between the Microbiota and *Anopheles* Mosquito Immune Response

Mosquitoes develop robust innate immune responses against invading pathogens such as viruses, bacteria (Levashina et al., 2001) and malaria parasites (Richman et al., 1997). The mosquito immune system is divided into two responses: humoral and cellular defense mechanisms.

In the humoral defense, the recognition of pathogens is done through the activation of effector molecules such as specific proteases that trigger processes such as melanization. Among these effectors, antimicrobial peptides of *An. gambiae* are known to be produced against different types of pathogens. These include defensin (active against Gram-positive bacteria), cecropin-1 (against Gram-positive and Gram-negative bacteria and fungi) and gambicin (against Gram-positive and Gram-negative bacteria) (Vizioli et al., 2000, 2001; Bartholomay and Michel, 2018).

The cellular immune responses are mediated by mosquito blood cells (hemocytes) and include phagocytosis of pathogens. Previous studies have shown that humoral and cellular immune responses involved three signaling pathways: the toll pathway (Luo and Zheng, 2000; Goto et al., 2003), the immunodeficiency pathway (IMD) (Meister et al., 2005) and janus kinase/signal transducer and activator of transcription (JAK/STAT) (Gupta et al., 2009). The *An. gambiae* JAK/STAT pathway contributes to anti-*Plasmodium* immune responses against the development of early *Plasmodium* spp. oocysts through the activation of nitric oxide synthase (NOS) (Gupta et al., 2009).

In general, the ability of mosquitoes to inhibit the growth of pathogens occurs through three mechanisms. Firstly, bacterial growth after a blood meal triggers an immune response *via* the immunodeficiency pathway (IMD), which causes the synthesis of antimicrobial peptides and other immune effectors (Meister et al., 2009). These effectors target bacterial populations in the mosquito midgut and exert antiparasitic effects (Meister et al., 2009). Secondly, symbiont bacteria of the *Anopheles* mosquito have been shown naturally to have the ability to inhibit *Plasmodium* spp. development. Specifically, the genus *Enterobacter* (Esp_Z) has been shown to inhibit *P. falciparum* ookinete, oocyst, and sporozoite formation in *An. gambiae* by up to 99% *via* elevated reactive oxygen species (ROS) synthesis (Cirimotich et al., 2011). Some mosquito gut bacteria, including *Serratia marcescens* and *Acinetobacter* spp. inhibit malaria parasite infection in mosquitoes (Wang et al., 2017). Thirdly, a microbiota-dependent immune priming system is reported during *Plasmodium* spp. infection. This effect protects mosquitoes from subsequent *Plasmodium* spp. infections and is likely mediated by hemocytes differentiation (Smith et al., 2015). Thus, insects’ gut microbiota appears as one of the important factors in host resistance to pathogen development. In particular, it has been shown that mosquito bacteria negatively impact oocysts density through colonization mechanisms involving either direct interactions between *Plasmodium* spp. and the microbiota or through induction of the mosquito’s immune response by the bacteria (Dong et al., 2009; Meister et al., 2009).

The presence of bacteria that naturally prevent *Plasmodium* infection in *Anopheles* mosquitoes provides a solid background for further genetic modification of predominant bacteria species found in these vectors to prevent parasite transmission. Indeed, it was already demonstrated that modified bacteria of the genus *Asaia* could express anti-malaria effectors, and the engineered strains obtained inhibit the development of malaria parasites (Shane et al., 2018).

## Microbiota-Mediated Insecticide Resistance in *Anopheles* Mosquitoes

Recently, several research reports showed associations between the mosquito microbiota and resistance to the current insecticides used for vector control. Recent work has identified 21 bacterial genera specific to the resistant *An. gambiae* harboring the *kdr*-East allele (L1014S, conferring resistance to permethrin) and 16 genera unique to the susceptible individuals (Omoke et al., 2021). Indeed, the well-known pyrethroid-degrading taxa bacteria of *Sphingobacterium*, *Lysinibacillus* and *Streptococcus* genera, in addition to the radiotolerant *Rubrobacter* were reported in resistant *An. gambiae* mosquitoes which were resistant at fivefold the diagnostic dose of permethrin (Omoke et al., 2021). Interestingly, the genus *Myxococcus*, was abundant in the susceptible *An. gambiae* and was not detected in their resistant counterparts (Omoke et al., 2021). *Ochrobactrum, Lysinibacillus*, and *Stenotrophomonas* genera (each of which comprised insecticide-degrading species) were significantly enriched in deltamethrin-resistant *An. coluzzii*, while susceptible mosquitoes had a significant reduction in bacterial diversity, with *Asaia* and *Serratia* as dominating microbial genera (Pelloquin et al., 2021). In the *Lysinibacillus* genus, *Lysinibacillus sphaericus* was demonstrated to be able to degrade up to 83% of cyfluthrin (a pyrethroid insecticide) by using the insecticide as a source of carbon or nitrogen (Hu et al., 2014).

An indirect effect of gut-associated microbiota on mosquito longevity by mediating insecticides resistance was also reported. *Klebsiella*, *Enterobacter*, *Staphylococcus* and *Aeromonas* were primarily found in the guts of the fourth instar larvae and non-blood fed adult females of the multiple-resistant *An. arabiensis* (Barnard et al., 2019). The organophosphate insecticides degrading bacterial species were found in fenitrothion resistant *An. albimanus*, including the predominant genera *Klebsiella*, *Enterobacter*, *Acinetobacter*, *Escherichia*, and *Salmonella* (Dada et al., 2018). Furthermore, pyrethroid insecticides exposure was found to induce changes in the cuticle, and the internal microbiota of both larvae and adults exposed *An. albimanus* (Dada et al., 2019). Indeed, *Pseudomonas fragi* and *Pseudomonas agglomerans* were more abundant in the internal microbiota of both alphacypermethrin- and permethrin-exposed *An. albimanus* adults, while unique taxa annotated as *Acinetobacter* and *Asaia* were more abundant in the internal and cuticle surface microbiota of non-exposed mosquitoes (Dada et al., 2019). In another vector species *An. stephensi*, four dominant genera, including *Pseudomonas*, *Aeromonas*, *Exiguobacterium*, and *Microbacterium*, were found in the midguts of temephos-resistant individuals (Soltani et al., 2017).

All these observations suggest that there is an additional microbe-mediated insecticide resistance mechanism in addition to the known target site modification mechanisms in these malaria vectors. The insecticide degrading properties could be due to the high expression of xenobiotic degrading genes and enzymes in bacteria when they are in contact with insecticidal molecules. Apart from larvicides that inter larvae through breeding water, insecticide molecules enter the adult mosquito body through the respiratory system (by inhalation) or the cuticle. Once the insecticides go through the cuticle, they enter the hemolymph where they could migrate to any organ of the insect. Thus, once in the gut, for example, they will be in contact with gut bacteria degrading insecticide. Then, the insecticide metabolism will lead to a decrease in the quantity of insecticidal molecules available to reach their target site. As a result, mosquitoes will be insensitive to that insecticide. However, further investigations are needed to understand the causality and pathways underlying such interactions. Yet, since microbes are likely to produce effector molecules that degrade insecticides, it is thought that the microbe-mediated mechanism of insecticide resistance would be likely of a metabolic nature (Dada et al., 2018; Omoke et al., 2021). This hypothesis is supported by other research findings. Indeed, high relative abundances of hydrolases, isomerases, and lyases were reported in xenobiotic-degrading bacteria colonizing fenitrothion resistant *An. albimanus* with two significantly enriched carboxylesterases (carboxymethylenebutenolidase and gluconolactonase), and two significantly enriched phosphomonoesterases (alkaline phosphatase, and acid phosphatase) (Dada et al., 2018).

A more thorough comprehension of the role of the microbiome in insecticide resistance will empower the improvement of techniques toward curbing the widespread resistance and sustaining the effectiveness of the current vector control interventions (Cansado-Utrilla et al., 2021). Several studies have reported significant differences in microbiota composition between susceptible and resistant mosquitoes, with the highest bacterial diversity found in susceptible mosquitoes (Dada et al., 2018; Barnard et al., 2019; Omoke et al., 2021). Thus, modulating mosquito bacterial diversity could lead to a change in insecticide resistance phenotype that would be beneficial for resistant vector populations’ management. In the actual context of the search for eco-friendly alternative vector control strategies, the success of malaria vector control requires a combined action of all existing control strategies. As a result, strategies targeting the microbiota can be considered to prevent insecticide degradation by mosquitoes’ bacteria.

## Conclusion

Mosquitoes have exceptionally diverse microbial taxa, which implies the significant effect of the microenvironment in modeling microbial organization. Several studies were carried out to characterize the wild mosquito microbiota. However, further research works are needed to better understand the full microbiome spectrum and its potential implications for malaria-transmitting vectors. More information on these vectors’ microbiota is fundamental for implementing paratransgenic strategies and symbiotic control approaches. In the current context of widespread insensitivity of malaria mosquitoes to the existing chemical insecticides, the role of bacteria in the mosquito immune system and resistance to insecticides open perspectives on the research of alternative biological control strategies based on endosymbiosis.

## Author Contributions

OD, AG, DN, and LSD contributed to the conception and design of the study. OD organized the sessions and wrote the first draft of the manuscript. OD, AM, AG, HS, HL, DN, LD, RB, PS, M-JF, RAg, RAk, and WM wrote sections of the manuscript. All authors contributed to manuscript revision, read, and approved the submitted version.

## Conflict of Interest

The authors declare that the research was conducted in the absence of any commercial or financial relationships that could be construed as a potential conflict of interest.

## Publisher’s Note

All claims expressed in this article are solely those of the authors and do not necessarily represent those of their affiliated organizations, or those of the publisher, the editors and the reviewers. Any product that may be evaluated in this article, or claim that may be made by its manufacturer, is not guaranteed or endorsed by the publisher.
